# Body Mass Index, High-Sensitivity C-Reactive Protein and Mortality in Chinese with Coronary Artery Disease

**DOI:** 10.1371/journal.pone.0135713

**Published:** 2015-08-17

**Authors:** Ding Ding, Min Wang, Dongfang Su, Changjiang Hong, Xinrui Li, Yunou Yang, Yuan Zhang, Gang Hu, Wenhua Ling

**Affiliations:** 1 Guangdong Provincial Key Laboratory of Food, Nutrition and Health, Department of Nutrition, School of Public Health, Sun Yat-sen University, Guangzhou, Guangdong, China; 2 Department of Cardiology, General Hospital of Guangzhou Military Command of People’s Liberation Army, Guangzhou, Guangdong, China; 3 Chronic Disease Epidemiology Laboratory, Pennington Biomedical Research Center, Baton Rouge, Louisiana, United States of America; Nagoya University, JAPAN

## Abstract

**Background:**

To investigate single and joint associations of body mass index (BMI) and serum high-sensitivity C-reactive protein (hsCRP) with death.

**Methods:**

The study included 1871 coronary artery disease (CAD) patients aged 40–85 year-old recruited from 2008 to 2011. Cox regression models were used to estimate the association of BMI and hsCRP with mortality. The data was analyzed in 2014.

**Results:**

During 3.1 years follow-up, 141 deaths were recorded, 110 died of cardiovascular disease (CVD). After adjustment of major CVD risk factors, there was a J-shaped association between BMI and all-cause and CVD mortality, and a positive association between hsCRP and mortality. The J-shaped association of BMI with mortality was present among patients who never smoked or with elevated hsCRP (≥3.0 mg/L). Compared with overweight (BMI 24–27.9 kg/m^2^) patients with normal hsCRP (<3.0 mg/L), obese patients (BMI≥28 kg/m^2^) with elevated hsCRP had a 3.41-fold risk of all-cause mortality (95% CI 1.49–7.80) and a 3.50-fold risk of CVD mortality (1.40–8.75), lean patients (BMI<24 kg/m^2^) with elevated hsCRP concentration had a 2.54-fold risk of all-cause mortality (1.36–4.74) and a 2.36-fold risk of CVD mortality (1.19–4.70).

**Conclusions:**

The association pattern between baseline BMI and mortality changed among different baseline hsCRP concentrations, indicating that low-grade inflammation may be related to BMI and secondary prognosis of CAD.

## Introduction

The prevalence of overweight and obesity has increased 27.5% for adults and 47.1% for children from 1980 to 2013 worldwide, and the number of overweight and obese individuals rises from 857 million in 1980 to 2.1 billion in 2013 [1]. Although this epidemic is most severe in developed countries, it is also causing a big public health challenge in developing countries such as China. Due to the rapid economic boom in the past decades, the accompanying changes in dietary habits and lifestyle lead to a significant increase of overweight and obese populations in China. More than 32% of Chinese adults aged 20 years and older were overweight and obese in 2013, and more than 345 million Chinese adults are projected to be overweight or obese by 2030 [1, 2]. Obesity is associated with increased risks of hypertension, diabetes, coronary artery disease (CAD), ischaemic stroke, and several kinds of cancer [3]. However, the association between BMI and mortality has been widely debated, especially for people with chronic diseases, such as diabetes, heart failure, and chronic kidney disease. BMI is shown to have a positive or U-shaped association with all-cause mortality in some epidemiological studies. Others find that obesity does not increase or even decrease mortality in some clinical conditions, thus the concept of “obesity paradox” is introduced. Among CAD patients, the association of BMI with mortality is still debated [4, 5].

In contrast, high-sensitivity C-reactive protein (hsCRP), a biomarker reflecting low-grade inflammation of body, has been identified as a risk factor for both CAD morbidity and secondary mortality [6, 7]. Some studies have shown that there is a strong positive association between hsCRP concentration and BMI, indicating obesity is somehow linked with low-grade inflammation [8]. Malnutrition- inflammation complex syndrome (MICS) may partly explain the existence of obesity paradox [9], and there is no report on the association of BMI and hsCRP with mortality among CAD patients. The aim of the present study was to evaluate the single and joint associations of BMI and serum hsCRP with the risk of all-cause and cardiovascular (CVD) mortality in Chinese CAD patients.

## Methods

### Participants

The recruitment of the Guangdong Coronary Artery Disease Cohort was between October 2008 and December 2011 [10, 11]. We enrolled 1980 successive patients admitted to the Cardiology Department of 3 superior specialty hospitals in Guangdong and diagnosed as CAD [International Classification of Diseases (ICD)-10 codes I20-I25] according to World Health Organization 1999/2000 guidelines [12, 13]. After excluding 109 participants with missing BMI or hsCRP measurements, the final sample comprised 1871 CAD patients aged 40 to 85 years. No differences in age (64.6 vs. 63.6 years) and percentage of men (65.1% vs. 65.6%) were found between excluded and retained participants. The study was approved by Sun Yat-sen University ethnic committee and all clinical investigation was conducted according to the principles expressed in the Declaration of Helsinki, and all participants signed the informed consent.

### Clinical measurements

A standardized questionnaire on general information of examination date, birth date, gender, education level, leisure-time physical activity, smoking habits, alcohol consumption, family history of CAD, medication history, and a validated food frequency questionnaire were conducted through a face-to-face interview. Smoking was defined as at least one cigarette a day and lasting more than six months. Alcohol drinking was defined as drinking any type of alcoholic beverage at least once a week and lasting more than six months. Smoking and drinking status was classified as never, past, or current.

Clinical characteristics, clinical tests’ results and treatment of participants were collected from an electronic case record system. At admission, trained nurses measured height, weight and blood pressure using a standard protocol. BMI was calculated by dividing weight in kilograms by the square of height in meters. Glomerular filtration rate (GFR) was used to assess renal function according to the most recent Modification of Diet in Renal Disease Study equation for standardized serum creatinine, which is estimated at GFR (eGFR) = 175 × (standardized serum creatinine in mg/dL) ^-1.154^ × Age^-0.203^ × 0.742 (if female). Degree of CAD was based on coronary artery stenosis degree of coronary angiography reports, which was categorized as not conduct, <50%, 50–74.9%, and ≥75%. Treatment information of CAD included percutaneous coronary intervention and coronary artery bypass graft. Venous blood samples were drawn in the next morning after hospital admission with at least 12 hours fasting. Lipids and fasting plasma glucose (FPG) were determined by standard methods immediately after collection. Blood samples were stored at -80°C until thawed and then analyzed. Serum hsCRP concentration was measured with a FlowCytomix technique using FlowCytomix Human Basic Kit (BMS8420FF, eBioscience, USA) together with Human CRP FlowCytomix Simplex Kit (BMS82288FF, eBioscience, USA) on a BD FACSCalibur instrument (BD Biosciences, USA). Data were obtained from CellQuest software (BD Biosciences, USA) and calculated by the FlowCytomix Program (eBioscience, USA). The limit of detection was 0.1mg/L, and the mean intra-assay coefficient of variation was 9%.

### Prospective follow-up

Follow-up data were collected from telephone contacts with patients or family members, reviewing hospitals’ medical records, and death registration of the Guangdong Provincial Center for Disease Control and Prevention. We collected patients’ health and disease conditions annually through telephone interviews and medical records. The surveys were followed to the end of July 2013 or patients’ death, whichever occurred first. Follow-up duration was calculated from the enrolment date. The ICD codes were used to code the cause of death, and the ICD codes I00-I99 were classified as CVD deaths. We set all-cause mortality and CVD mortality as our primary outcomes.

### Statistical analysis

We began the data analyses in 2014. Differences in normally distributed continuous variables between men and women were analyzed by the general linear model after adjustment for age. A chi-square test was used for categorical variables and a Kruskal-Wallis one-way ANOVA was used for hsCRP. The associations of baseline BMI and serum hsCRP with the risks of all-cause and CVD mortality were analyzed by Cox proportional hazards models. BMI was evaluated in the following 2 ways: (1) as 4 categories (underweight <18.5, normal weight 18.5–23.9, overweight 24–27.9 [reference group], and obese ≥28 kg/m^2^), and (2) as a continuous variable. Baseline hsCRP concentration was log-transformed and then classified into quartiles. All analyses were adjusted for age and gender, and further for education, leisure-time physical activity, smoking, alcohol drinking, and then additionally history of hypertension, diabetes, dyslipidemia, and use of antihypertensive, anti-diabetic, cholesterol-lowering, and anti-platelet drugs. hsCRP was included in the final models when evaluating BMI with mortality, and BMI was included in the final models when evaluating hsCRP with mortality. Since the interactions between gender and BMI levels on the risks of all-cause and CVD mortality were not statistically significant, data for men and women were combined in the analyses to maximize the statistical power. To avoid the potential bias due to severe diseases at baseline, additional analyses were carried out excluding the subjects who died during the first year of follow-up. We used restricted cubic splines in Cox models to test whether there is a dose-response or non-linear association of BMI as a continuous variable with all-cause and CVD mortality risk. Statistical significance was considered to be P<0.05. All statistical analyses were performed with IBM SPSS Statistics 20.0 (IBM SPSS Inc, Chicago, III), and SAS for Windows, version 9.3 (SAS Institute, Cary, NC).

## Results

### Baseline characteristics of participants

At baseline, only age, blood pressure, high-density lipoprotein cholesterol, eGFR, history of hypertension and dyslipidemia, and use of antihypertensive drugs were different among BMI categories (S1 Table).

### Associations of BMI with mortality

**Fig 1:**
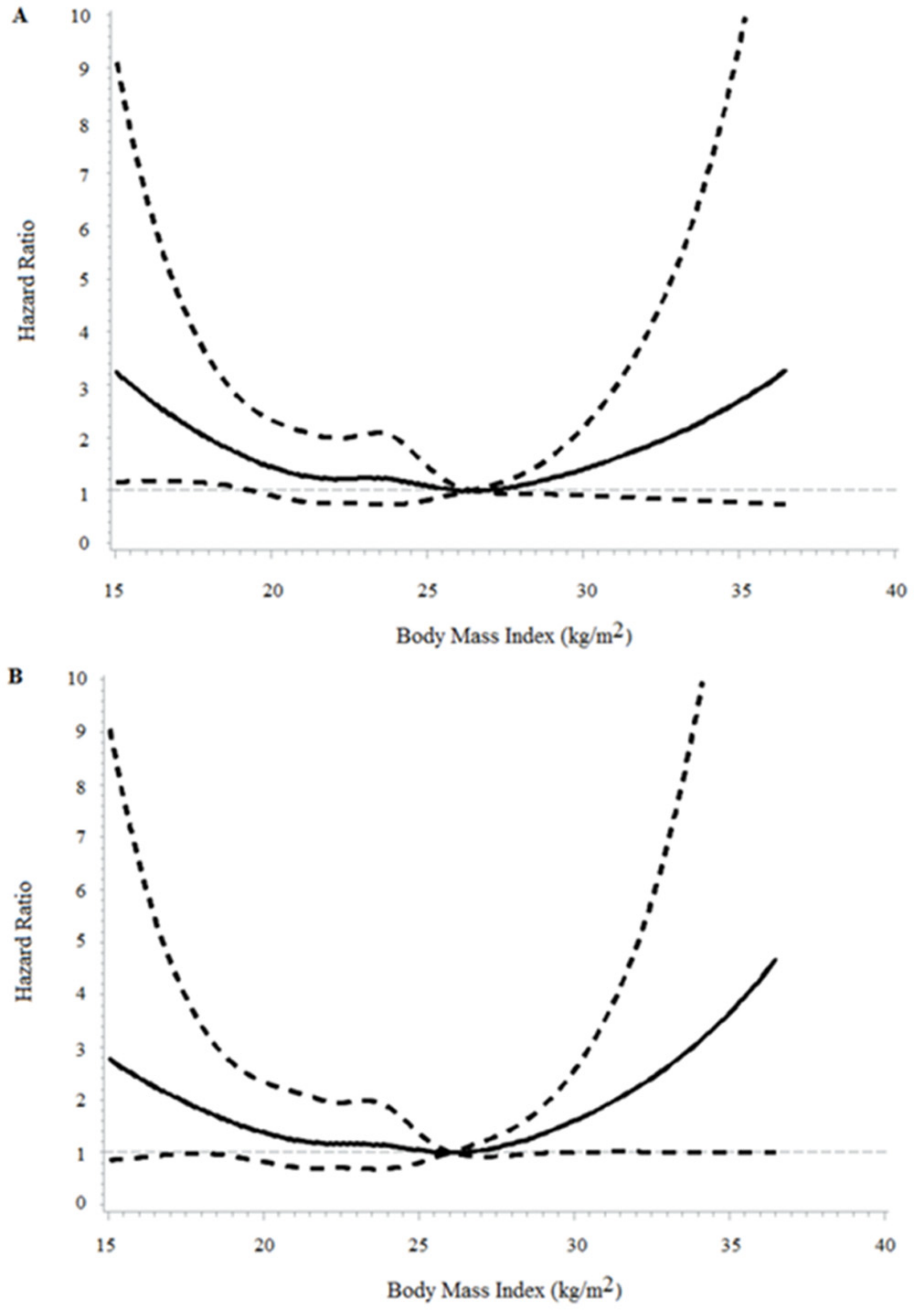
Spline plots displaying the risk of all-cause (A) and cardiovascular (B) mortality over the range of body mass index. Adjusted for age, gender, education, leisure–time physical activity, smoking, alcohol drinking, history of hypertension, diabetes, dyslipidemia, use of antihypertensive drugs, anti-diabetic drugs, cholesterol-lowering drugs, anti-platelet drugs, and high-sensitivity C-reactive protein. Dashed lines represented 95% CI.

During a median follow-up of 3.1 years, 141 deaths were recorded, 110 of which were due to CVD. The multivariable-adjusted Model 1 (age, gender, education, leisure-time physical activity, smoking, and alcohol drinking) hazard ratios (HRs) across four BMI categories (<18.5, 18.5–23.9, 24–27.9, ≥28 kg/m^2^) were 2.22 (95% CI 1.18–4.18), 1.50 (95% CI 1.01–2.24), 1.00, and 2.18 (95% CI 1.18–4.05) for all-cause mortality, and 2.00 (95% CI 0.96–4.17), 1.42 (95% CI 0.90–2.22), 1.00, and 2.44 (95% CI 1.25–4.78) for CVD mortality (Table 1). After further adjustment for other CVD risk factors (history of hypertension, diabetes, dyslipidemia, and use of antihypertensive drugs, anti-diabetic drugs, cholesterol-lowering drugs, and anti-platelet drugs, multivariable-adjusted Model 2) and serum hsCRP concentration (multivariable-adjusted Model 3), this J-shaped association did not change. When BMI was considered as a continuous variable by using restricted cubic splines, a nadir of the J-shaped association of BMI with all-cause and CVD mortality was observed at BMI of 24–28 kg/m^2^ (Fig 1).

**Table 1:** Hazard ratios for all-cause and cardiovascular mortality according to body mass index category.

	Baseline BMI category (kg/m^2^)				*P*_difference_
	Underweight (<18.5)	Normal (18.5–23.9)	Overweight (24–27.9)	Obesity (≥28)	
No. of subjects	88	906	687	190	
Person-years	248	2807	2142	589	
All-cause mortality					
No. of death	14	76	36	15	
Adjustment for age and gender	2.46 (1.32–4.59)	1.54 (1.04–2.30)	1.00	2.06 (1.12–3.79)	0.01
Multivariable adjustment					
Model 1^a^	2.22 (1.18–4.18)	1.50 (1.01–2.24)	1.00	2.18 (1.18–4.05)	0.02
Model 2^b^	2.32 (1.23–4.39)	1.45 (0.96–2.17)	1.00	2.06 (1.10–3.85)	0.03
Model 3^c^	2.37 (1.25–4.48)	1.45 (0.96–2.18)	1.00	2.05 (1.09–3.84)	0.02
Cardiovascular mortality					
No. of death	10	58	29	13	
Adjustment for age and gender	2.21 (1.07–4.56)	1.46 (0.93–2.28)	1.00	2.25 (1.16–4.35)	0.04
Multivariable adjustment					
Model 1^a^	2.00 (0.96–4.17)	1.42 (0.90–2.22)	1.00	2.44 (1.25–4.78)	0.04
Model 2^b^	2.16 (1.03–4.53)	1.37 (0.87–2.17)	1.00	2.35 (1.18–4.67)	0.04
Model 3^c^	2.22 (1.05–4.66)	1.37 (0.87–2.17)	1.00	2.35 (1.18–4.66)	0.04

^a^ Model 1 was adjusted for age, gender, education, leisure-time physical activity, smoking, and alcohol drinking.

^b^ Model 2 was adjusted for model 1 covariates plus history of hypertension, diabetes, dyslipidemia, and use of antihypertensive drugs, anti-diabetic drugs, cholesterol-lowering drugs, and anti-platelet drugs.

^c^ Model 3 was adjusted for model 2 covariates plus high-sensitivity C-reactive protein.

BMI, Body mass index.

### Associations of CRP with mortality

Baseline serum hsCRP was positively associated with all-cause and CVD mortality. After adjustment for major CVD risk factors, the HRs across quartiles of hsCRP in the multivariable-adjusted Model 2 were 1.00, 2.65 (95% CI 1.38–5.07), 4.01 (95% CI 2.17–7.44), and 3.16 (95% CI 1.68–5.94) for all-cause mortality (*P*_trend_ <0.001), and 1.00, 3.61 (95% CI 1.64–7.98), 4.57 (95% CI 2.11–9.88), and 4.19 (95% CI 1.92–9.13) for CVD mortality (*P*_trend_ = 0.001), respectively (Table 2).

**Table 2:** Hazard ratios for all-cause and cardiovascular mortality according to different concentration of high-sensitivity C-reactive protein.

	Baseline high-sensitivity C-reactive protein				*P*_trend_
	Quartile 1	Quartile 2	Quartile 3	Quartile 4	
No. of subjects	468	466	469	468	
Person-years	1550	1476	1379	1380	
All-cause mortality					
No. of death	13	34	49	45	
Adjustment for age and gender	1.00	2.66 (1.40–5.03)	3.98 (2.16–7.35)	3.38 (1.82–6.27)	<0.001
Multivariable adjustment					
Model 1^a^	1.00	2.46 (1.29–4.67)	3.81 (2.06–7.04)	3.07 (1.65–5.72)	<0.001
Model 2^b^	1.00	2.65 (1.38–5.07)	4.01 (2.17–7.44)	3.16 (1.68–5.94)	<0.001
Cardiovascular mortality					
No. of death	8	29	36	37	
Adjustment for age and gender	1.00	3.68 (1.68–8.06)	4.74 (2.2–10.21)	4.52 (2.01–9.72)	0.001
Multivariable adjustment					
Model 1^a^	1.00	3.36 (1.53–7.36)	4.44 (2.06–9.58)	4.07 (1.89–8.79)	0.002
Model 2^b^	1.00	3.61 (1.64–7.98)	4.57 (2.11–9.88)	4.19 (1.92–9.13)	0.001

^a^ Model 1 was adjusted for age, gender, education, leisure-time physical activity, smoking, and alcohol drinking.

^b^ Model 2 was adjusted for model 1 covariates plus body mass index, history of hypertension, diabetes, dyslipidemia, and use of antihypertensive drugs, anti-diabetic drugs, cholesterol-lowering drugs, anti-platelet drugs.

### Joint associations of BMI and hsCRP with mortality

**Fig 2:**
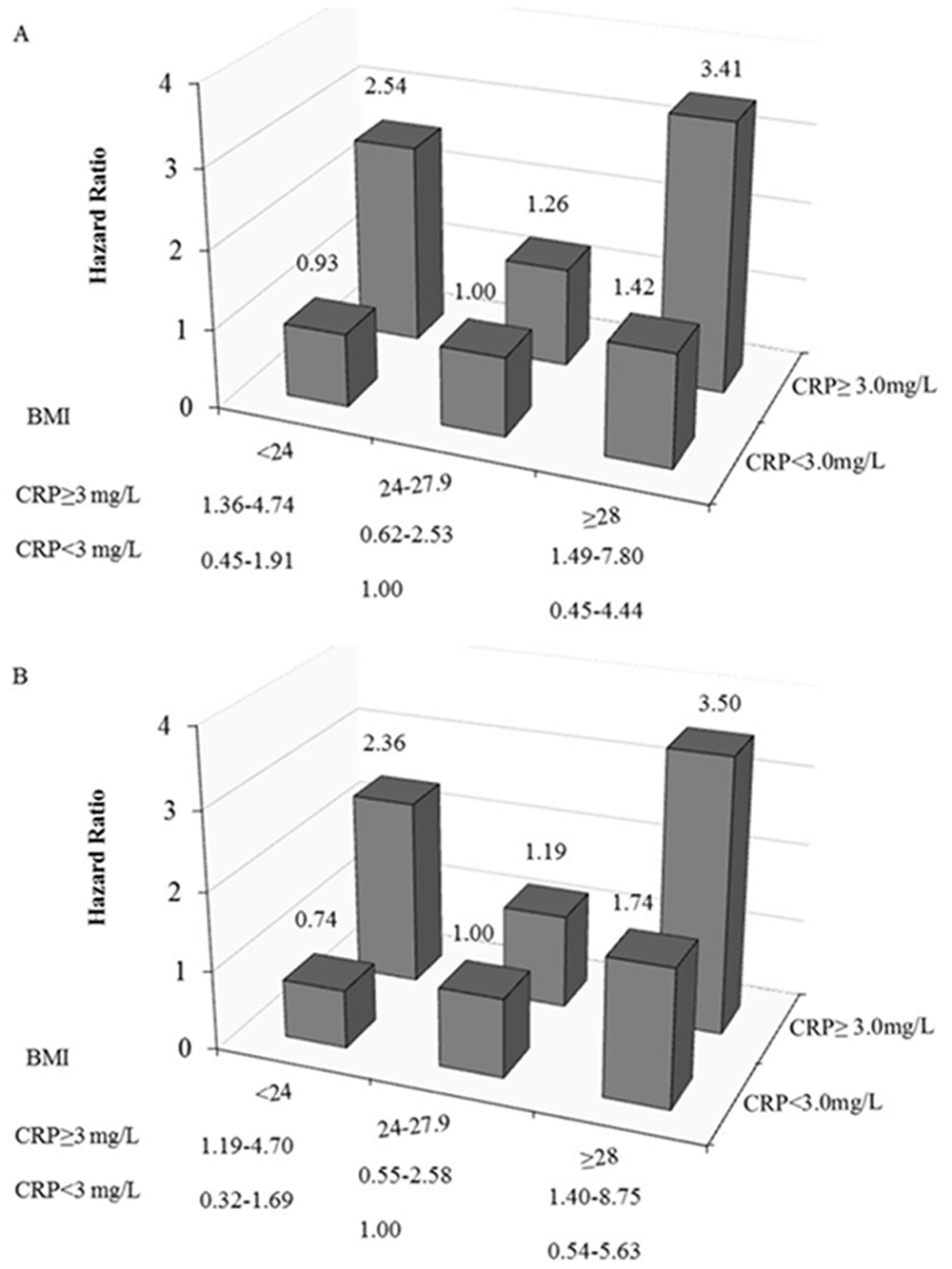
Hazard ratios of all-cause (A) and cardiovascular (B) mortality according to different levels of body mass index and C-reactive protein. Adjusted for age, gender, education, leisure–time physical activity, smoking, and alcohol drinking. 95% confidential intervals of hazard ratios were shown under the figure. hsCRP = high-sensitivity C-reactive protein.

Fig 2 showed HRs of all-cause and CVD mortality for the joint effects of BMI and serum hsCRP. We used 3 categories of BMI (lean <24, overweight 24–27.9, and obese ≥28 kg/m^2^) and 2 categories of hsCRP (normal hsCRP <3, and elevated hsCRP ≥3 mg/L) [14]. The J-shaped associations between BMI and the risks of all-cause and CVD mortality were still present among patients with elevated hsCRP concentration (≥3mg/L), while the association of BMI and mortality changed to a positive trend among patients with normal hsCRP concentration (<3mg/L). Compared with overweight patients with normal hsCRP concentration, obese patients with elevated hsCRP concentration had a 3.41-fold risk of all-cause mortality (95% CI 1.49–7.80) (Fig 2A) and a 3.50-fold risk of CVD mortality (95% CI 1.40–8.75) (Fig 2B), lean patients with elevated hsCRP concentration had a 2.54-fold risk of all-cause mortality (95% CI 1.36–4.74) (Fig 2A) and 2.36-fold risk of CVD mortality (95% CI 1.19–4.70) (Fig 2B).

### Sensitivity analyses

When we did an additional analysis among patients who never smoked, the J-shaped association of BMI with all-cause and CVD mortality was confirmed among non-smokers (S2 Table). When we excluded the participants who died during the first year of follow-up (n = 61), the J-shaped association did not change. Patients with BMI of 18.5–23.9 kg/m^2^ and BMI ≥28 kg/m^2^ had significantly increased risks of all-cause and CVD mortality (data not shown).

## Discussion

This is the first study assessing the association of BMI and serum hsCRP with all-cause and CVD mortality in patients with established CAD. We found a J-shaped association between baseline BMI and the risks of all-cause and CVD mortality among Chinese CAD patients, while baseline hsCRP was positively associated with death risk. This J-shaped association was only observed among patients with elevated hsCRP concentration. For patients with normal hsCRP concentration, the risk of death increased with BMI levels.

Obesity has been found to be associated with increased risks of cardiometabolic diseases such as hypertension, hyperlipidemia, diabetes and CAD among general populations. However, the association of obesity with death risk is inconsistent among people with chronic diseases [3, 4]. The Diaphane Collaborative Study Group in France is the first one to report that the risk of overall mortality decreases with increasing BMI among hemodialysis patients [15]. This has been referred to as “obesity paradox”. Subsequently, several studies have shown that obesity paradox might be present in other clinical conditions including diabetes [16], hypertension [17], and heart failure [18]. But among CAD patients, the association of BMI with mortality is contradictory. Many different studies have reported positive associations [19], inverse associations [20], J-shaped associations [21], or no associations [22]. In the present study, underweight (BMI<18.5 kg/m^2^) and obese (BMI≥28 kg/m^2^) patients were at a higher risk of all-cause and CVD mortality, while overweight patients offered protection against mortality. This J-shaped association between BMI and mortality is also confirmed by three large CAD cohorts of Canada, America and Sweden, and the increased mortality is only observed in patients with extreme obesity (BMI ≥40 kg/m^2^) [5, 21, 23]. The difference in the significantly increased mortality among underweight and obese CAD patients from above and our studies may be related to different races. A previous study on obesity paradox among end-stage renal disease patients also found a U-shaped association between BMI and mortality among Asian Americans, while this association changed to be inverse among whites [24]. Comparing with Caucasians, Asian patients have a much lower mean BMI. In the present study, the prevalence of obesity is 10.2%, however, it is up to 36.2% in the American cohort [21].

Unlike the debated association between obesity and mortality, the detrimental effect of underweight on CAD patients has been shown by many epidemiologic studies, although the mechanism is not clear [4]. Previous studies on maintenance dialysis patients suggest that chronic inflammation may causally tie low weight to increased mortality through malnutrition. This is referred to as the MICS [25]. It is widely accepted that inflammation plays a crucial role in both initiation and progression of CAD, and circulating hsCRP is believed to be a critical risk marker for CAD [26, 27]. We have also found a positive association between serum hsCRP and mortality among Chinese CAD patients. This indicates that the acute-phase inflammatory process may play a harmful role in the prognosis of CAD patients [6, 7]. Some investigators indicate that inflammation, especially the state of acute-phase response, may promote catabolic processes, which stimulate protein degradation and suppression of protein synthesis. Besides, inflammation can also induce anorexia. Both these effects may promote protein-energy malnutrition, and thus decrease BMI [28–30]. This is also observed among patients with cardiac disease [31]. In the present study, a J-shaped association between BMI and mortality was found among CAD patients with elevated hsCRP concentration, but this association changed to be positive among those with normal hsCRP concentration. This seems to agree with the explanation of malnutrition- inflammation complex syndrome. On the other hand, we found that overweight offered protection against all-cause and CVD mortality only among patients with elevated hsCRP concentration, but this protective function disappeared among patients with normal hsCRP concentration. The cause of the benefit of overweight has been unknown. From the present results, we suppose that this protection may probably be associated with some degree of inflammation. Although inflammation is considered as a key factor in all stages of CAD, from initiation through progression, and, ultimately, the thrombotic complications, inflammation is originally a biological immune response to remove the injurious stimuli and to initiate the healing process [26, 32]. And it is known that adipose tissue secretes several inflammatory factors [33], so mild inflammation induced by overweight may be beneficial to CAD patients. Whether there is a paradox of inflammation with CAD remains a question. In addition to obesity, there are many paradoxical phenomena such as reactive oxygen species (ROS), as large fundamental studies have shown that elevated ROS possesses both pathogenetic and physiological effects of living system, and the mechanism remains unsolved [34]. Therefore, more studies including experimental research, randomized controlled trials and perspective studies with large sample are needed to evaluate the interrelationships between BMI, inflammation, and adverse outcomes in CAD patients.

There are several limitations in the present study. First, our subjects were enrolled from hospitals which may bring selection bias. In general, in-patients are considered to have a more severe disease status than non-hospitalized people. However, we included both acute CAD patients and those with stable manifestation, and some of them were electively admitted patients with mild status. Thus we can reduce the bias. Second, we used BMI only to measure obesity of patients. Although BMI is the most generally used epidemiological measurement of obesity, it could not directly distinguish central from peripheral adiposity. Third, we only measured BMI and hsCRP at baseline and did not further measure BMI during follow-up.

In conclusion, we found a J-shaped association between baseline BMI and death risk among Chinese CAD patients, with the lowest risk of mortality among overweight patients. We also confirmed the positive association between baseline serum hsCRP and mortality in CAD patients. However, the association pattern changed among different hsCRP concentration, indicating that low-grade inflammation may be related to BMI and secondary prognosis of CAD.

## Supporting Information

S1 Table Baseline characteristics by body mass index category among coronary artery disease patients. (DOCX)

S2 Table Hazard ratios for all-cause and cardiovascular mortality according to body mass index category among subpopulations. (DOCX)
