# Supplementary material for: Body Mass Index, High-Sensitivity C-Reactive Protein and Mortality in Chinese with Coronary Artery Disease
Source: PLoS One. 2015 Aug 17;10(8):e0135713. doi: 10.1371/journal.pone.0135713 (PMC4539189; doi:10.1371/journal.pone.0135713)
Supplement: S1 Table — (DOCX) [file pone.0135713.s001.docx]

**Table S1. Baseline characteristics by body mass index category among coronary artery disease patients^a^**

| Characteristic | Baseline BMI category (kg/m^2^) | | | | *P*_difference_ |
| --- | --- | --- | --- | --- | --- |
|  | Underweight (<18.5) | Normal  (18.5-23.9) | Overweight  (24-27.9) | Obesity  (≥28) |  |
| N (%) | 88 (4.7) | 906 (48.4) | 687 (36.7) | 190 (10.2) |  |
| Age at baseline (yrs) | 67.6 (1.2) | 64.2 (0.4) | 63.1 (0.4) | 60.4 (0.8) | <0.001 |
| Male (%) | 62.5 | 66.9 | 66.1 | 59.5 | 0.23 |
| Systolic blood pressure (mm Hg) | 124 (2.4) | 131 (0.7) | 136 (0.8) | 142 (1.6) | <0.001 |
| Diastolic blood pressure (mm Hg) | 70 (1.3) | 75 (0.4) | 77 (0.5) | 81 (0.9) | <0.001 |
| Fasting plasma glucose (mmol/L) | 6.36 (0.29) | 6.34 (0.09) | 6.66 (0.10) | 6.62 (0.20) | 0.11 |
| Low-density lipoprotein cholesterol (mmol/L) | 2.74 (0.11) | 2.95 (0.03) | 3.00 (0.04) | 3.07 (0.07) | 0.06 |
| High-density lipoprotein cholesterol (mmol/L) | 1.20 (0.03) | 1.10 (0.01) | 1.06 (0.01) | 1.04 (0.02) | <0.001 |
| High-sensitivity C-reactive protein (mg/L) | 4.76 (0.94-12.1) | 3.39 (0.89-13.6) | 3.95 (1.15-13.9) | 4.80 (1.35-13.1) | 0.34 |
| Years of education (%) |  |  |  |  | 0.59 |
| ≤9 | 69.4 | 60.8 | 61.2 | 58.4 |  |
| 10-12 | 15.3 | 21.7 | 19.1 | 24.0 |  |
| ≥13 | 15.3 | 17.5 | 19.7 | 17.6 |  |
| Leisure-time physical activity (%) |  |  |  |  | 0.17 |
| None | 41.8 | 36.4 | 30.7 | 39.6 |  |
| <30 minutes/day | 16.4 | 19.8 | 24.2 | 21.4 |  |
| ≥30 minutes/day | 41.8 | 43.8 | 45.1 | 39.0 |  |
| Smoking (%) |  |  |  |  | 0.81 |
| Never | 58.1 | 60.5 | 58.5 | 61.4 |  |
| Past | 5.8 | 9.5 | 9.3 | 9.0 |  |
| Current | 36.1 | 30.0 | 32.2 | 29.6 |  |
| Alcohol drinking (%) |  |  |  |  | 0.37 |
| Never | 75.7 | 78.6 | 77.4 | 72.0 |  |
| Past | 4.3 | 7.2 | 7.7 | 7.3 |  |
| Current | 20.0 | 14.2 | 14.9 | 20.7 |  |
| Coronary stenosis extent in coronary angiography |  |  |  |  | 0.17 |
| Not conduct | 35.8 | 26.0 | 30.9 | 34.7 |  |
| <50% | 6.0 | 15.0 | 14.2 | 12.3 |  |
| 50-74.9% | 10.4 | 8.6 | 7.8 | 5.9 |  |
| ≥75% | 47.8 | 50.4 | 47.1 | 47.1 |  |
| Estimated glomerular filtration rate (mL/min/1.73m^2^), (%) |  |  |  |  | <0.001 |
| ≥90 | 26.4 | 28.3 | 29.2 | 32.6 |  |
| 60-89 | 39.1 | 50.4 | 49.1 | 39.8 |  |
| 30-59 | 25.3 | 19.3 | 19.9 | 23.6 |  |
| 15-29 | 4.6 | 1.5 | 1.5 | 3.4 |  |
| <15 | 4.6 | 0.5 | 0.3 | 0.6 |  |
| History of diseases (%) |  |  |  |  |  |
| Hypertension | 46.6 | 52.3 | 59.7 | 67.9 | <0.001 |
| Diabetes | 18.2 | 22.5 | 25.8 | 34.2 | 0.003 |
| Dyslipidemia | 14.8 | 25.7 | 32.8 | 35.8 | <0.001 |
| Use of medication before admission (%) |  |  |  |  |  |
| Antihypertensive drugs | 37.5 | 43.6 | 53.6 | 57.2 | <0.001 |
| Anti-diabetic drugs | 9.1 | 15.9 | 16.7 | 18.2 | 0.26 |
| Lipid-lowering drugs | 6.8 | 11.8 | 12.5 | 13.9 | 0.38 |
| Anti-platelet drugs | 11.4 | 21.1 | 19.3 | 18.2 | 0.15 |

^a^ Continuous data are reported as mean (standard error) if normally distributed and median (25th, 75th percentile) if non-normally distributed, categorical data are reported as percentages. All continuous variables are adjusted for age and gender, except for age (adjusted for sex only). CAD, coronary artery disease.
