# Supplementary material for: Body Mass Index, High-Sensitivity C-Reactive Protein and Mortality in Chinese with Coronary Artery Disease
Source: PLoS One. 2015 Aug 17;10(8):e0135713. doi: 10.1371/journal.pone.0135713 (PMC4539189; doi:10.1371/journal.pone.0135713)
Supplement: S2 Table — (DOCX) [file pone.0135713.s002.docx]

**Table S2. Hazard ratios for all-cause and cardiovascular mortality according to body mass index category among subpopulations**

|  | Baseline BMI category (kg/m^2^) | | | | *P*_difference_ |
| --- | --- | --- | --- | --- | --- |
|  | Underweight (<18.5) | Normal (18.5-23.9) | Overweight (24-27.9) | Obesity (≥28) |  |
| **Never smoker** |  |  |  |  |  |
| No. of subjects | 50 | 544 | 402 | 116 |  |
| Person-years | 146 | 1716 | 1279 | 359 |  |
| All-cause mortality |  |  |  |  |  |
| No. of death | 7 | 48 | 22 | 12 |  |
| Multivariable adjustment^a^ | 2.04 (0.85-4.89) | 1.45 (0.87-2.41) | 1.00 | 2.68 (1.31-5.50) | 0.04 |
| Cardiovascular mortality |  |  |  |  |  |
| No. of death | 5 | 36 | 18 | 10 |  |
| Multivariable adjustment^a^ | 1.84 (0.67-5.09) | 1.28 (0.72-2.27) | 1.00 | 2.70 (1.22-5.95) | 0.08 |
| **Ever or current smoker** |  |  |  |  |  |
| No. of subjects | 38 | 362 | 285 | 74 |  |
| Person-years | 101 | 1091 | 863 | 230 |  |
| All-cause mortality |  |  |  |  |  |
| No. of death | 7 | 28 | 14 | 3 |  |
| Multivariable adjustment^a^ | 2.32 (0.90-5.98) | 1.5 (0.78-2.87) | 1.00 | 1.05 (0.29-3.78) | 0.32 |
| Cardiovascular mortality |  |  |  |  |  |
| No. of death | 5 | 22 | 11 | 3 |  |
| Multivariable adjustment^a^ | 2.22 (0.74-6.66) | 1.59 (0.76-3.33) | 1.00 | 1.41 (0.38-5.26) | 0.48 |

^a^ Model was adjusted for age, gender, education, leisure-time physical activity, and alcohol drinking.
